# Mapping opportunities for the earlier diagnosis of psoriasis in primary care settings in the UK: results from two matched case–control studies

**DOI:** 10.3399/BJGP.2022.0137

**Published:** 2022-10-04

**Authors:** Maha Abo-Tabik, Rosa Parisi, Catharine Morgan, Sarah Willis, Christopher EM Griffiths, Darren M Ashcroft

**Affiliations:** Centre for Dermatology Research, Division of Musculoskeletal & Dermatological Sciences, University of Manchester; National Institute for Health and Care Research (NIHR) Manchester Biomedical Research Centre, Manchester.; Division of Informatics, Imaging & Data Sciences, University of Manchester; NIHR Manchester Biomedical Research Centre, Manchester.; Division of Population Health, Health Services Research & Primary Care, University of Manchester; NIHR Manchester Biomedical Research Centre, Manchester.; Division of Innovation Management and Policy, Alliance Manchester Business School, University of Manchester, Manchester.; Centre for Dermatology Research, Division of Musculoskeletal & Dermatological Sciences, University of Manchester; Dermatology Centre, Salford Royal NHS Foundation Trust, Manchester.; NIHR Greater Manchester Patient Safety Translational Research Centre and Division of Pharmacy and Optometry, University of Manchester; NIHR Manchester Biomedical Research Centre, UK.

**Keywords:** diagnosis, general practice, primary care, psoriasis

## Abstract

**Background:**

The diagnosis of psoriasis may be missed or delayed in primary care settings.

**Aim:**

To examine trends in healthcare events before a diagnosis of psoriasis.

**Design and setting:**

Two matched case–control studies using electronic healthcare records delineated from the Clinical Practice Research Datalink (CPRD GOLD and Aurum) in the UK.

**Method:**

Individuals aged ≥18 years with an incident diagnosis of psoriasis (case group) between 1 January 2010 and 29 December 2017 were identified and matched by age, sex, and general practice with six individuals without psoriasis (control group). Healthcare activities were examined and annual incidence rates and incidence rate ratios (IRRs) with 95% confidence intervals (CIs) for 10 years before the index date were compared between case and control groups.

**Results:**

There were 17 320 people with psoriasis and 99 320 controls included from CPRD GOLD, and 11 442 people with psoriasis and 65 840 controls extracted from CPRD Aurum. Data from CPRD GOLD showed that people with psoriasis were up to eight times more likely to be diagnosed with pityriasis rosea at 6 months (IRR 7.82, 95% CI = 4.09 to 14.95) before the index date than the control group. The case group were twice as likely to be diagnosed with eczema (IRR 1.90, 95% CI = 1.76 to 2.05) or tinea corporis (IRR 1.99, 95% CI = 1.74 to 2.27) 1 year before the index date. The case group were more likely to report dry skin, rash, skin texture changes, and itching than the control group up to 5 years before the index date. The most frequently reported clinical feature was rash with an IRR of 2.71 (95% CI = 2.53 to 2.92) at 1 year before the index date. The case group were prescribed topical corticosteroids (IRR 1.97, 95% CI = 1.88 to 2.07) or topical antifungals (IRR 1.92, 95% CI = 1.78 to 2.07) in the year before the index date twice as often as those in the control group.

**Conclusion:**

Findings suggest that the diagnosis of psoriasis may be missed or delayed in a UK primary care setting for up to 5 years for some individuals, hence leading to a potentially detrimental delay in establishing an appropriate treatment regimen.

## INTRODUCTION

Psoriasis is a systemic, inflammatory, long-term disease with characteristic clinical signs.^1^ It affects the quality of life of affected individuals to a substantial degree;^2^ however, its overall effect often extends beyond the skin, being associated with other medical conditions such as psoriatic arthritis,^3^ cardiovascular disease,^4^ respiratory diseases,^5^ liver disease,^6^ and depression.^7^ Disease progression in psoriasis is unpredictable in that some patients have mild disease that is stable for many years, whereas, for others, it quickly progresses to moderate-to-severe disease.^2^ Thus, psoriasis is a complex health problem that requires a comprehensive care approach for both early diagnosis and treatment. The global prevalence of psoriasis is estimated to be 0.59% in adults and about 0.47% in the overall population,^7^ and psoriasis affects around 3% of the general population in the UK.^8^

Epidemiological studies suggested that the burden of psoriasis is greater in high-income countries of North America and Europe than in other regions.^9^ In 2014, the World Health Organization (WHO) highlighted that many people in the world suffer needlessly from psoriasis because of an incorrect or delayed diagnosis. The WHO also emphasised the psychological and pathological consequences of a delayed or incorrect diagnosis of psoriasis and recognised this as a global concern.^10^^,^^11^

Present treatment approaches are aimed at providing individualised care that focuses on improving the signs and symptoms of the rash while proactively screening for and treating any associated comorbidities.^12^ More recently, increasing efforts are being made to trial the impact of early intervention targeting complete clearance, which may improve control of psoriasis and may also modify disease course and burden.^13^ To address this, psoriasis needs to be recognised early.

The impact of the delay in the diagnosis and treatment of psoriatic arthritis has previously been investigated and it has been suggested that a ≥6-month delay in this diagnosis and initiation of treatment is associated with deterioration in patients’ quality of life in comparison with shorter periods.^14^ In the case of psoriasis, a multicentre international observational study reported on the diagnostic delay and estimated this to be 1.6 years (standard deviation 4.8) on average.^15^

**Table:** How this fits in

Many people with psoriasis experienced missed or delayed diagnosis. Primary care professionals are most often the first point of contact for people with psoriasis. The diagnosis of psoriasis can be a challenging task for non-dermatologists. In this study, examining electronic health records from general practices showed that people who eventually ended with psoriasis diagnosis have an increased frequency of GP consultations from 5 years before their diagnosis of psoriasis is documented. Individuals with psoriasis are often prescribed topical corticosteroids and/or topical antifungal medications before being diagnosed with psoriasis. These medications may mask the signs of psoriasis.

The diagnosis of psoriasis relies on the identification of clinical features, which are incorporated into clinical diagnostic criteria.^16^ However, its variable clinical presentation and resemblance to other skin conditions (for example, eczema and tinea corporis) make it difficult to recognise, especially in those populations where access to specialist dermatology care is restricted, which may result in missed or delayed diagnosis.

In many countries, including the UK, primary care professionals represent the first point of contact for people with dermatological conditions including psoriasis.^17^ Despite being one of the most commonly seen skin conditions in a primary care setting, there is a lack of studies on missed or delayed diagnosis of psoriasis. The primary aim of this study was to examine the electronic health records (EHRs) of individuals with and without psoriasis and to investigate incident rates of other differential diagnoses, characteristic clinical features, and treatments.

## METHOD

### Data source, participants, and study design

Data from the Clinical Practice Research Datalink (CPRD) were used in the study. The CPRD is one of the largest databases of longitudinal health records from primary care in the world. Data from CPRD are divided into two databases depending on the information technology system the general practice uses for their patient management. General practices using Vision software contribute to CPRD Gold^18^ and those using EMIS Web software contribute to CPRD Aurum.^19^ CPRD GOLD has contributing general practices from across the UK whereas CPRD Aurum collects anonymised health records from general practices predominately in England. The population coverage of the dataset is considered to be representative in terms of age, sex, ethnicity, and geographical distributions of the UK general population.^18^^,^^19^

The study is reported in line with the recommendations of the RECORD statement.^20^ This case–control study was conducted using de-identified EHRs that were extracted by a third party and anonymised before being made available for research purposes. Thus, no patient consent was required.

Participants were included as ‘incident cases’ of psoriasis diagnosed between 1 January 2010 and 29 December 2017. Each individual with psoriasis was matched with six eligible individuals without a diagnosis of psoriasis. The matching was undertaken on calendar time (index date of cases), the exact year of birth, sex, and registered general practice.

### Clinical events of interest

An a priori list of clinical events that could potentially be related to missed opportunities for psoriasis diagnosis in primary care settings was identified. Clinical events of interest were grouped into three categories:

- differential diagnosis for psoriasis;
- clinical features; and
- prescribed medications (Supplementary Table S1).

Diagnoses and clinical features were identified by Read codes. The list of clinical events was reviewed by one of the authors, who is an experienced dermatologist, to ensure clinical relevance to the aims and objectives of the study.

#### Differential diagnosis

Potential differential diagnoses of psoriasis included seborrheic dermatitis, other eczema (including contact dermatitis, atopic dermatitis, neurodermatitis, discoid eczema, asteatotic eczema, and hand dermatitis), tinea corporis, candidal dermatoses, and pityriasis rosea.

#### Clinical features

The number of times that people in the case group and control group consulted their GP with clinical features that were considered suggestive of psoriasis was examined. Clinical features that may precede a diagnosis of psoriasis included itching, dry skin, rash, and changes in skin texture (scale, plaque, and crust).

#### Prescribed medications

Two groups of medications that are often prescribed for people before their diagnoses of psoriasis is confirmed were identified. These included topical corticosteroids and topical antifungals from the *British National Formulary* (BNF).^21^

### Data analysis

Descriptive statistics were used to calculate the median and interquartile range (IQR) for demographic characteristics. Age at index date, sex, geographical region at the general practice level, and socioeconomic status based on the Index of Multiple Deprivation (IMD) were used as a measure of socioeconomic deprivation of residential neighbourhood,^22^^–^24 which was linked at the general practice level. The frequency of GP visits was also considered. Absolute numbers (that is, total numbers in the case and control groups) and their frequency (or proportion) were used to report on demographic data analysis.

Incidence rates and 95% confidence intervals (CIs) were calculated for each clinical event (differential diagnosis, clinical features, and prescribed medication) per 1000 person–years for each year within 10 years before the index date for individuals with and without psoriasis. The incidence rate ratios (IRRs) and 95% CIs were also calculated for each clinical event at 6 months, 1 year, 3 years, and 5 years before index date for the case and control groups.

## RESULTS

### CPRD GOLD dataset

#### Demographic characteristics

The study population was extracted from 796 participating GP practices. In total, 17 320 individuals with incident diagnosis of psoriasis were identified from CPRD GOLD and matched to 99 320 individuals without a psoriasis diagnosis. The baseline demographic characteristics are described in Table 1. Median age at index date was 51 (IQR 36–64) and 50 (IQR 36–64) years for the case and control groups, respectively; sex was 52.18% female and 47.82% male in both groups.

**Table 1.** Baseline demographic characteristics of the study population (CPRD GOLD)

**Total**	**Case group (*n* = 17 320)**	**Control group (*n* = 99 320)**
**Sex, *n* (%)**		
Male	8282 (47.82)	47 491 (47.82)
Female	9038 (52.18)	51 829 (52.18)

**Age at index, years, median (IQR)**	51 (36–64)	50 (36–64)

**Region, *n* (%)**		
London	2457 (14.19)	14 023 (14.12)
South England	7227 (41.73)	41 626 (41.91)
Midlands and East England	3831 (22.12)	21 924 (22.07)
North England	3805 (21.97)	21 747 (21.90)

**Number of GP consultations before the**		
**index date, median (IQR)**		
4–5 years before index date	7 (2–13)	5 (2–12)
3–4 years before index date	8 (3–15)	6 (2–12)
2–3 years before index date	8 (3–16)	6 (2–13)
1–2 years before index date	10 (5–18)	8 (4–15)
0–1 year before index date	11 (5–19)	8 (4–15)

**Socioeconomic status, IMD quintile, *n* (%)^a^**		
1 (least deprived)	4020 (23.21)	23 997 (24.16)
2	3830 (22.11)	22 405 (22.56)
3	3422 (19.76)	19 553 (19.69)
4	3343 (19.30)	18 775 (18.90)
5 (most deprived)	2695 (15.56)	14 533 (14.63)

*CPRD = Clinical Practice Research Datalink. IMD = Index of Multiple Deprivation. IQR = interquartile range.*

a *Missing data: cases 10 (0.06%) and controls 57 (0.06%).*

#### Frequency of GP consultations

Overall, individuals with psoriasis were more likely to visit their GP than those without psoriasis (Table 1). Visits to the GP practices for individuals with psoriasis increased during the 5-year period before the index date by almost 60%, from a median of 7 (IQR 2–13) per year at 5 years before the index date to 11 (IQR 5–19) visits per year at 1 year before the index date. In comparison, the frequency of GP consultations for those without psoriasis showed a less noticeable increase from 5 (IQR 2–12) to 8 (IQR 4–15) over the same 5-year period before the index date.

#### Differential diagnosis

People in the case group were more likely to receive a diagnosis of pityriasis rosea, eczema, seborrheic dermatitis, tinea corporis, and candidal dermatoses than those in the comparator group from 5 years before the index date. The incidence rates of being diagnosed with one of the aforementioned skin conditions were markedly higher for the case group than those without psoriasis in the final year before the index date, as shown in Table 2.

**Table 2.** IRRs of clinical events recorded 6 months, 1, 3, and 5 years before the index date (CPRD GOLD)

**Clinical events**	**6 months, IRR (95% CI)**	**1 year, IRR (95% CI)**	**3 years, IRR (95% CI)**	**5 years, IRR (95% CI)**
**Seborrheic dermatitis**	2.34 (1.82 to 3.00)	1.97 (1.65 to 2.35)	1.49 (1.33 to 1.66)	1.27 (1.13 to 1.38)
**Eczema**	2.23 (1.99 to 2.50)	1.90 (1.76 to 2.05)	1.41 (1.35 to 1.48)	1.23 (1.18 to 1.28)
**Tinea corporis**	2.52 (2.09 to 3.03)	1.99 (1.74 to 2.27)	1.43 (1.32 to 1.56)	1.25 (1.17 to 1.34)
**Candida skin infections**	1.46 (1.32 to 1.74)	1.44 (1.29 to 1.61)	1.28 (1.20 to 1.37)	1.15 (1.08 to 1.21)
**Pityriasis rosea**	7.82 (4.09 to 14.95)	3.24 (2.24 to 5.27)	1.71 (1.28 to 2.27)	1.38 (1.09 to 1.75)
**Dry skin**	2.05 (1.54 to 2.72)	1.52 (1.24 to 1.86)	1.38 (1.22 to 1.57)	1.08 (1.06 to 1.30)
**Rash**	4.00 (3.62 to 4.41)	2.71 (2.53 to 2.92)	1.63 (1.55 to 1.71)	1.32 (1.27 to 1.38)
**Skin texture changes**	2.17 (1.69 to 2.29)	1.55 (1.39 to 1.77)	1.23 (1.14 to 1.31)	1.13 (1.06 to 1.20)
**Itching**	1.39 (1.00 to 1.93)	1.54 (1.22 to 1.94)	1.26 (1.10 to 1.45)	1.18 (1.05 to 1.32)
**Topical corticosteroids**	2.58 (2.39 to 2.79)	1.97 (1.88 to 2.07)	1.46 (1.42 to 1.50)	1.24 (1.21 to 1.27)
**Topical antifungal treatment**	2.32 (2.08 to 2.59)	1.92 (1.78 to 2.07)	1.43 (1.36 to 1.49)	1.24 (1.20 to 1.29)

*CPRD = Clinical Practice Research Datalink. IRR = incidence rate ratio.*

**Figure 1.**
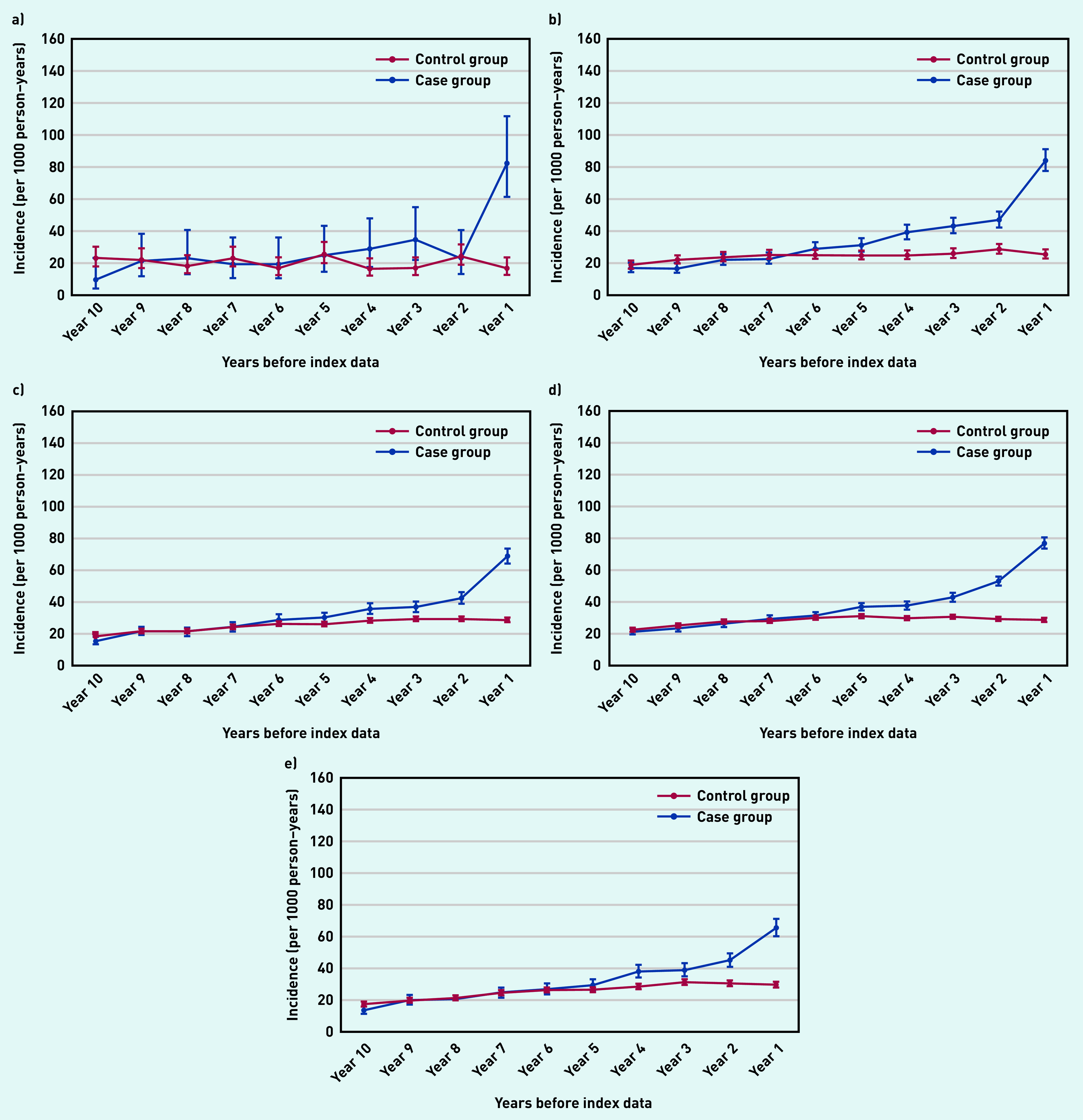
*Annual incidence rate per 1000 person– years of diagnosing a) pityriasis rosea; b) seborrheic dermatitis; c) tinea corporis; d) eczema; and e) candida skin infection from 10 years before the index date. Bars are 95% confidence intervals (CPRD GOLD). Eczema includes contact dermatitis, atopic dermatitis, neurodermatitis, discoid eczema, asteatotic eczema, and hand dermatitis. CPRD = Clinical Practice Research Datalink.*

Individuals with psoriasis were almost eight times more likely to be diagnosed with pityriasis rosea (Figure 1a) and twice as likely to be diagnosed with seborrheic dermatitis (Figure 1b) and eczema (Figure 1d) within the year before the index date than those in the control group.

In addition, individuals with psoriasis were 2.5 times more likely to be diagnosed with tinea corporis (Figure 1c) and 1.5 times more likely to be diagnosed with candidal dermatoses (Figure 1e) in the final year before the index date than those without psoriasis.

#### Clinical features

People with psoriasis more frequently reported rash, dry skin, and skin texture changes (including scales, plaque, and crust) than those without a psoriasis diagnosis before the index date, as shown in Table 2.

**Figure 2.**
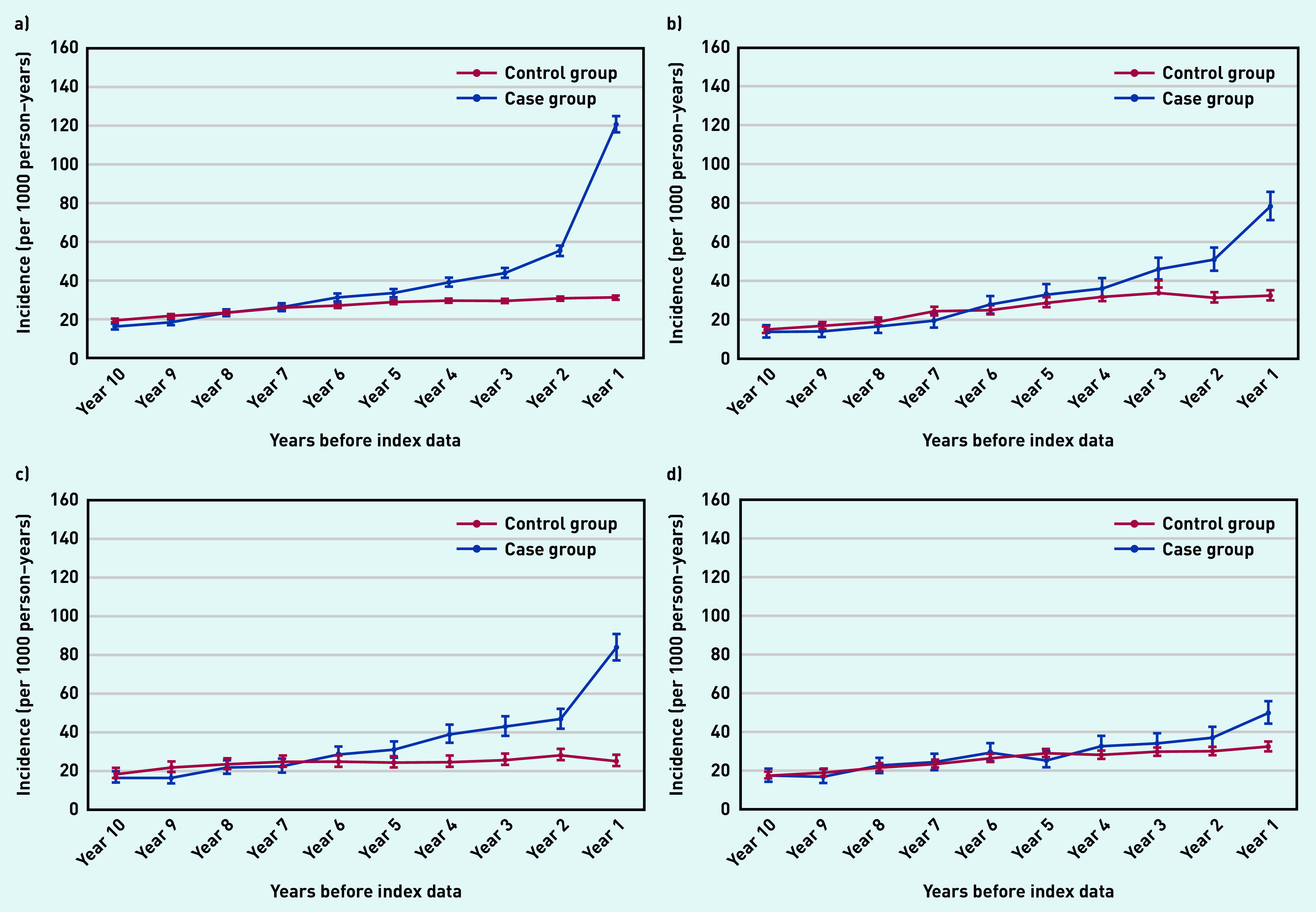
*Annual incidence rate per 1000 person–years of recording clinical features suggestive of psoriasis from 10 years before the index date. a) Rash; b) dry skin; c) skin texture changes; and d) itching. Bars are 95% confidence intervals (CPRD GOLD). CPRD = Clinical Practice Research Datalink.*

The most frequently reported clinical feature was skin rash (Figure 2a). Those who ended up with a psoriasis diagnosis were four times more likely to report skin rash in the final year before the index date than those in the control group.

People in the case group were twice as likely to report dry skin (Figure 2b) and skin texture changes (Figure 2c) in the final year before the index date than those in the control group.

Individuals in the control group were only slightly more likely to report itching than those in the control group (Figure 2d).

#### Prescribed medications

There was an increasing likelihood of a person in the case group being prescribed topical corticosteroids or topical antifungal medication closer to the index date compared with the control group, as shown in Table 2.

**Figure 3.**
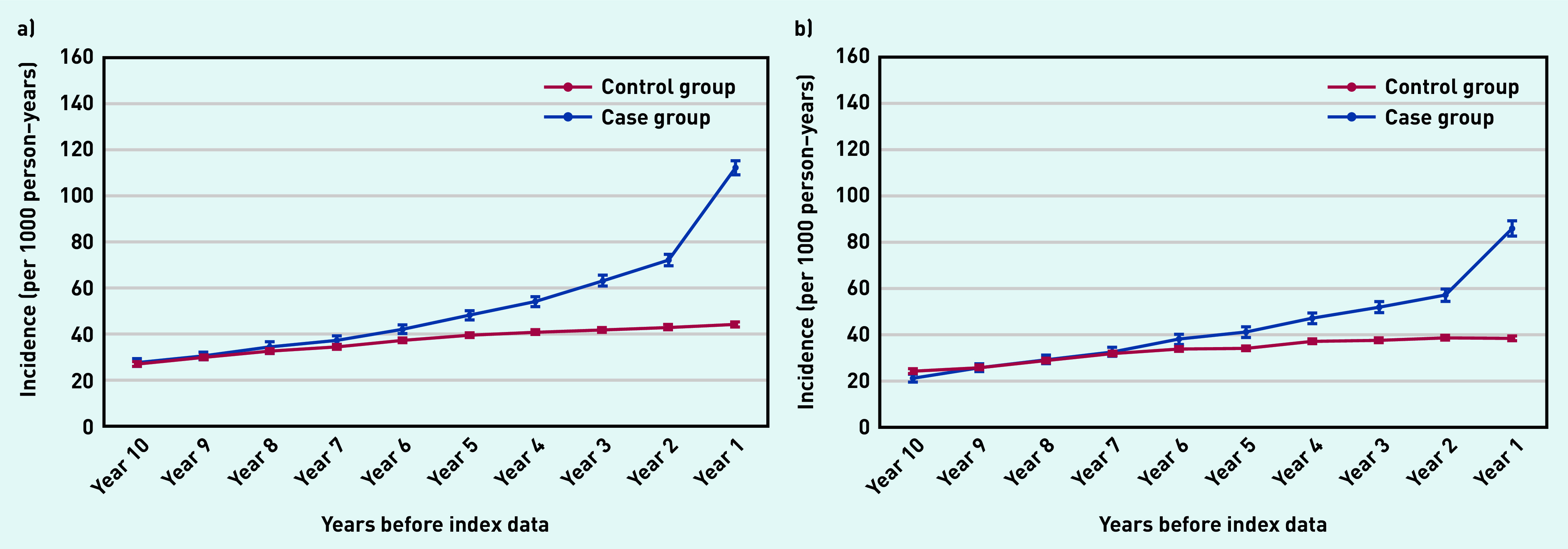
*Annual incidence rate per 1000 person–years of prescribing a) topical corticosteroids; and b) topical antifungals from 10 years before the index date. Bars are 95% confidence intervals (CPRD GOLD). CPRD = Clinical Practice Research Datalink.*

Those with a confirmed psoriasis diagnosis were almost twice as likely to be prescribed topical corticosteroids (Figure 3a) or topical antifungals (Figure 3b) within the final year before the index date (that is, date of documented psoriasis diagnosis) than those in the control group. Closer to the index date (at 6 months before a confirmed psoriasis diagnosis), people in the case group were 2.5 and 2.3 times more likely to receive topical corticosteroids and topical antifungal medication, respectively, than those in the control group.

The IRR for all investigated clinical events at 6 months, 1 year, 3 years, and 5 years before the index date is shown in Table 2.

### CPRD Aurum dataset

Data from CPRD Aurum showed similar findings to CPRD GOLD. In total, 11 442 people with an incident psoriasis diagnosis and 65 840 without psoriasis were included in this study. The baseline demographic characteristics of the study cohort are shown in Supplementary Table S2. The median age was 50 (IQR 35–64) years for both the case and control groups. The female:male ratio was similar for the case and control groups with almost 52% female and 48% male patients (cases = male 8282, female 9038; controls = male 47 491, female 51 829). The study population was extracted from 176 GP practices contributing to the CPRD Aurum database.

The frequency of GP consultations increased steadily from seven visits in 5 years before the index date to 12 visits in the final year before the index date, as shown in Supplementary Table S2.

Trends for incidence rates for the examined clinical events (differential diagnosis, clinical features, and prescribed medication) were all similar to the findings from CPRD GOLD. The annual incidence rate for the investigated possible missed clinical events are shown in Supplementary Figures S1–S3. The IRRs are shown in Supplementary Table S3.

## DISCUSSION

### Summary

To the authors’ knowledge, this is the first study to retrospectively analyse data collected from medical records to investigate primary care consultations before a diagnosis of psoriasis. This has resulted in the identification of premonitory clinical events that could potentially be related to a diagnosis of psoriasis before it is made. The current study found that people with psoriasis were more likely to visit general practices than those without psoriasis from 5 years before the index date.

Patients in the case group had higher chances of being diagnosed with skin conditions other than psoriasis before the index date (that is, date of confirmed psoriasis diagnosis) than those in the control group. Such skin conditions included pityriasis rosea, eczema, and/or fungal infections. Additionally, the study found higher reporting of symptoms related to psoriasis such as rash, dry skin, and skin texture changes in individuals who eventually developed psoriasis than in those without psoriasis.

Individuals with psoriasis were more likely to be prescribed a topical corticosteroid or antifungal medication before a documented diagnosis of psoriasis than were patients in the control group. The frequent use of these medications could mask signs and symptoms of psoriasis and contribute to further delay in diagnosis.

All the examined healthcare events (that is, differential diagnosis, clinical features, and prescribed medications) tend to increase through the 5 years before the index date among people with psoriasis. Hence, this suggests possible delays in psoriasis diagnosis of up to 5 years for some individuals.

### Strengths and limitations

The main strength of the study is that two independent case–control studies were conducted using primary care EHRs (CPRD GOLD and CPRD Aurum) and similar findings are reported in the two studies. Additionally, data recorded in the EHR are recorded prospectively thereby minimising the risk of recall bias.

The main limitation of this study is that only individuals aged ≥18 years were included, thus missed opportunities for psoriasis diagnosis among younger individuals at risk were not explored.

The other limitation is the lack of comparison across skin of colour. Psoriasis presents differently on different skin colours^1^^,^^16^^,^^25^ and the lack of experience in recognising psoriasis on darkly pigmented skin could potentially be a factor in a missed or delayed diagnosis.

### Comparison with existing literature

Studies on the clinical diagnosis of psoriasis have been limited. The authors’ recent international e-Delphi study undertaken by the Global Psoriasis Atlas reached consensus on a set of clinical examination-based diagnostic criteria for chronic plaque psoriasis.^16^ These diagnostic criteria were developed to facilitate psoriasis case recognition by non-dermatologists (such as primary healthcare professionals) in an attempt to improve psoriasis diagnosis in settings where access to specialist dermatology care was restricted. In this e-Delphi study the different clinical presentation of psoriasis across wide ethnic backgrounds and different affected body sites (that is, different psoriasis clinical phenotypes) was also captured.

### Implications for research and practice

Missing a diagnostic opportunity implies that an alternative approach could have enabled an earlier and correct diagnosis.^26^ For the practising clinician, data from this study raise awareness regarding a missed diagnosis or misdiagnosis of psoriasis among primary care physicians and emphasises the need to improve non-dermatologists’ diagnostic skills for psoriasis via specifically designed training courses (for example, online training tools) to encourage them to follow the consensus-agreed diagnostic criteria when suspecting a diagnosis of psoriasis.^16^

Physicians should consider the possibility of psoriasis in people with the following medical history and symptoms and signs of a skin condition:

- frequent GP consultations for their skin-related complaints;
- received a diagnosis of seborrheic dermatitis, other eczema (including contact dermatitis, atopic dermatitis, neurodermatitis, discoid eczema, asteatotic eczema, and hand dermatitis), tinea corporis, candida skin infections, and/or pityriasis rosea in the past;
- frequently reporting itching, dry skin, rash, and skin texture changes (scale, plaque, and crust); and
- being prescribed topical corticosteroids and/or topical antifungal medication with no or minimal improvement.

Timely diagnosis of psoriasis may promote early targeted, person-specific treatment, and screening for and treatment of comorbidities thereby improving disease course and burden, and reducing the chance of cumulative life-course impairment.^12^^,^^27^^,^^28^ Nevertheless, early diagnosis of psoriasis could also encourage changes to a healthier lifestyle. Lifestyle changes such as weight loss, reducing alcohol intake, and smoking cessation have been suggested as possible favourable psoriasis disease-course modifiers.^29^^,^^30^

The findings from the current study show that people with psoriasis have an increased number of healthcare interactions for several years before a diagnosis of psoriasis is made. These results support the need to investigate further whether a missed opportunity for diagnosis of chronic plaque psoriasis could be prevented by following expert-agreed diagnostic criteria for the condition.^16^

Furthermore, future work might be needed to explore the pre-diagnostic period of psoriasis, using data from secondary care settings.

## Data

This study used anonymised patient-level data from the Clinical Practice Research Datalink (CPRD) that are not publicly available because of confidentiality considerations. However, researchers can access CPRD’s databases by contacting the Medicines and Healthcare products Regulatory Agency. Details of the application process and conditions of access are available at https://www.cprd.com/Data-access.
